# Methyl 2-(but-3-en­yl)-4-hy­droxy-1,1-dioxo-2*H*-1λ^6^,2-benzothia­zine-3-carboxyl­ate

**DOI:** 10.1107/S1600536812022908

**Published:** 2012-05-31

**Authors:** Muhammad Nadeem Arshad, Islam Ullah Khan, Muhammad Zia-ur-Rehman, Muhammad Danish, K. Travis Holman

**Affiliations:** a Department of Chemistry, University of Gujrat, Gujrat 50781, Pakistan; b Materials Chemistry Laboratory, Department of Chemistry, GC University, Lahore 54000, Pakistan; c Applied Chemistry Research Centre PCSIR Laboratories Complex, Lahore 54600, Pakistan; d Department of Chemistry, Georgetown University, 37th and ‘O’ Streets NW Washington, DC 20057-1227, USA

## Abstract

In the title compound, C_14_H_15_NO_5_S, the thia­zine ring adopts a sofa conformation and an intra­molecular O—H⋯O hydrogen bond forms an *S*(6) ring. In the crystal, molecules are linked *via*C—H⋯O inter­actions.

## Related literature

For the synthesis, see: Arshad *et al.* (2011*b* ▶); Zia-ur-Rehman, *et al.* (2006 ▶). For related structures, see: Arshad *et al.* (2011*a* ▶, 2012 ▶). For graph-set notation, see: Bernstein *et al.* (1995 ▶).
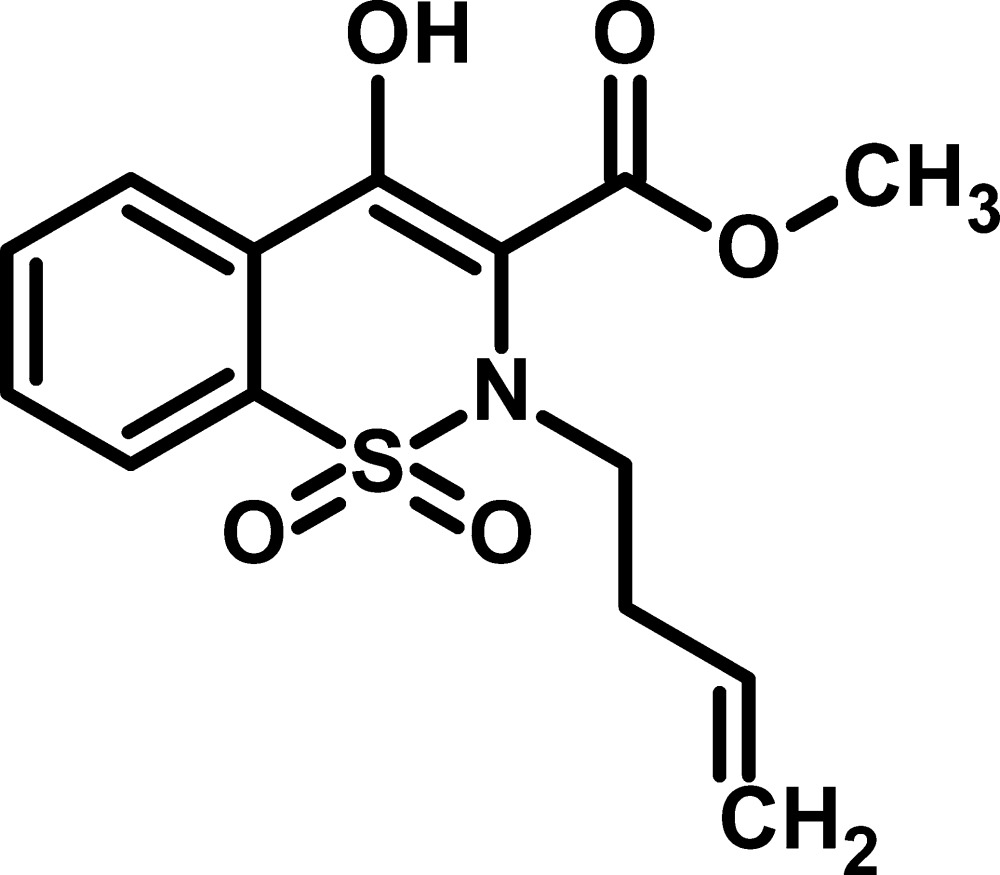


## Experimental

### 

#### Crystal data

C_14_H_15_NO_5_S
*M* *_r_* = 309.33
Orthorhombic, 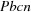

*a* = 25.265 (8) Å
*b* = 8.929 (3) Å
*c* = 12.584 (4) Å
*V* = 2839.0 (15) Å^3^
*Z* = 8
Mo *K*α radiation
μ = 0.25 mm^−1^
*T* = 100 K
0.41 × 0.36 × 0.19 mm

#### Data collection

Bruker SMART 1K diffractometer
Absorption correction: multi-scan (*SADABS*; Bruker, 2001 ▶) *T*_min_ = 0.905, *T*_max_ = 0.954
22868 measured reflections
3445 independent reflections
2643 reflections with *I* > 2σ(*I*)
*R*_int_ = 0.081

#### Refinement

*R*[*F* ^2^ > 2σ(*F* ^2^)] = 0.042
*wR*(*F* ^2^) = 0.109
*S* = 1.05
3445 reflections
194 parameters
H atoms treated by a mixture of independent and constrained refinement
Δρ_max_ = 0.33 e Å^−3^
Δρ_min_ = −0.52 e Å^−3^

### 

Data collection: *SMART* (Bruker, 2001 ▶); cell refinement: *SAINT* (Bruker, 2001 ▶); data reduction: *SAINT*; program(s) used to solve structure: *SHELXS97* (Sheldrick, 2008 ▶); program(s) used to refine structure: *SHELXL97* (Sheldrick, 2008 ▶); molecular graphics: *PLATON* (Spek, 2009 ▶) and *X-SEED* (Barbour 2001 ▶); software used to prepare material for publication: *WinGX* (Farrugia, 1999 ▶) and *PLATON*.

## Supplementary Material

Crystal structure: contains datablock(s) I, global. DOI: 10.1107/S1600536812022908/bt5926sup1.cif

Structure factors: contains datablock(s) I. DOI: 10.1107/S1600536812022908/bt5926Isup2.hkl

Supplementary material file. DOI: 10.1107/S1600536812022908/bt5926Isup3.cml

Additional supplementary materials:  crystallographic information; 3D view; checkCIF report

## Figures and Tables

**Table 1:** Hydrogen-bond geometry (Å, °)

*D*—H⋯*A*	*D*—H	H⋯*A*	*D*⋯*A*	*D*—H⋯*A*
C4—H4⋯O2^i^	0.95	2.52	3.269 (2)	136
O1—H1*O*⋯O4	0.84 (3)	1.81 (3)	2.577 (2)	151 (3)

Symmetry code: (i) 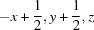.

**Fig. 1.**
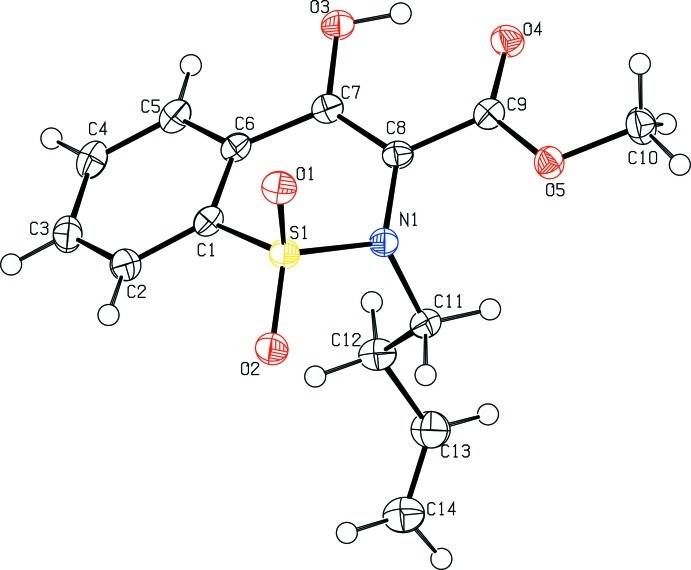
Molecular structure of the title compound with displacement ellipsoids drawn at the 50% probability.

## supplementary crystallographic information

### Comment

Herein we report the crystal structure of the title compound in continuation of our research on the synthesis (Arshad *et al.*, 2011*b*), biological activities (Zia-ur-Rehman *et al.*, 2006) and crystal structures (Arshad *et al.*, 2011*a*, 2012) of thiazine related heterocyles.

The title compound is the *N*-3-butenyl derivative of methyl-4-hydroxy-2*H*-1,2-benzothiazine-3-carboxylate 1,1-dioxide. The planar methyl ester moiety (r.m.s deviation 0.008Å) is oriented at dihedral angles of 11.39 (10)° and 16.97 (11)° with respect to the thiazine (C1/C6/C7/C8/N1/S1) and benzene (C1/C2/C3/C4/C5/C6) rings. The thiazine ring adopts a sofa conformation and is inclined at 17.27 (12)° with respect to the benzene ring. A six membered (C7/O1/H1O/O4/C9/C8) ring with graph set notation S^1^_1_(6) (Bernstein *et al.*, 1995) is formed through an O—H···O intramolecular hydrogen bond. The crystal structure is stabilized by weak C—H···O interactions (Tab. 1).

### Experimental

The synthesis of the titled compound has been published (Arshad *et al.*, 2011*a*). The title compound was recrystallized from ethylacetate under slow evaporation of the solvent.

### Refinement

The H-atoms bonded to C were positioned with idealized geometry with C—H ranging from 0.95 Å to = 0.99Å and refined using a riding model with *U*_iso_(H) = 1.5 *U*_eq_(C) for methyl and *U*_iso_(H) = 1.2 *U*_eq_(C) for the remaining H atoms. The coordinates of the H atom bonded to O were refined with *U*_iso_(H) 1.5*U*_eq_(O).

### Crystal data

**Table:**

C_14_H_15_NO_5_S	*F*(000) = 1296
*M**_r_* = 309.33	*D*_x_ = 1.447 Mg m^−^^3^
Orthorhombic, *P**b**c**n*	Mo *K*α radiation, λ = 0.71073 Å
Hall symbol: -P 2n 2ab	Cell parameters from 4741 reflections
*a* = 25.265 (8) Å	θ = 2.4–27.3°
*b* = 8.929 (3) Å	µ = 0.25 mm^−^^1^
*c* = 12.584 (4) Å	*T* = 100 K
*V* = 2839.0 (15) Å^3^	Block, colorless
*Z* = 8	0.41 × 0.36 × 0.19 mm

### Data collection

**Table:**

Bruker SMART 1K diffractometer	3445 independent reflections
Radiation source: fine-focus sealed tube	2643 reflections with *I* > 2σ(*I*)
Graphite monochromator	*R*_int_ = 0.081
φ and ω scans	θ_max_ = 28.3°, θ_min_ = 2.4°
Absorption correction: multi-scan (*SADABS*; Bruker, 2001)	*h* = −33→32
*T*_min_ = 0.905, *T*_max_ = 0.954	*k* = −11→11
22868 measured reflections	*l* = −16→16

### Refinement

**Table:**

Refinement on *F*^2^	Primary atom site location: structure-invariant direct methods
Least-squares matrix: full	Secondary atom site location: difference Fourier map
*R*[*F*^2^ > 2σ(*F*^2^)] = 0.042	Hydrogen site location: inferred from neighbouring sites
*wR*(*F*^2^) = 0.109	H atoms treated by a mixture of independent and constrained refinement
*S* = 1.05	*w* = 1/[σ^2^(*F*_o_^2^) + (0.0432*P*)^2^ + 1.393*P*] where *P* = (*F*_o_^2^ + 2*F*_c_^2^)/3
3445 reflections	(Δ/σ)_max_ = 0.001
194 parameters	Δρ_max_ = 0.33 e Å^−^^3^
0 restraints	Δρ_min_ = −0.52 e Å^−^^3^

### Special details

**Table:**

Geometry. All e.s.d.'s (except the e.s.d. in the dihedral angle between two l.s. planes) are estimated using the full covariance matrix. The cell e.s.d.'s are taken into account individually in the estimation of e.s.d.'s in distances, angles and torsion angles; correlations between e.s.d.'s in cell parameters are only used when they are defined by crystal symmetry. An approximate (isotropic) treatment of cell e.s.d.'s is used for estimating e.s.d.'s involving l.s. planes.
Refinement. Refinement of *F*^2^ against ALL reflections. The weighted *R*-factor *wR* and goodness of fit *S* are based on *F*^2^, conventional *R*-factors *R* are based on *F*, with *F* set to zero for negative *F*^2^. The threshold expression of *F*^2^ > σ(*F*^2^) is used only for calculating *R*-factors(gt) *etc*. and is not relevant to the choice of reflections for refinement. *R*-factors based on *F*^2^ are statistically about twice as large as those based on *F*, and *R*- factors based on ALL data will be even larger.

### Fractional atomic coordinates and isotropic or equivalent isotropic displacement parameters (Å^2^)

**Table:**

	*x*	*y*	*z*	*U*_iso_*/*U*_eq_	
S1	0.383769 (17)	0.36570 (5)	0.46460 (3)	0.01998 (13)	
O1	0.28657 (5)	0.22258 (15)	0.20949 (10)	0.0233 (3)	
O2	0.38708 (5)	0.20923 (14)	0.48963 (10)	0.0237 (3)	
O3	0.41184 (5)	0.47125 (15)	0.52964 (10)	0.0252 (3)	
O4	0.37180 (5)	0.18207 (15)	0.10068 (10)	0.0242 (3)	
O5	0.44900 (5)	0.25535 (14)	0.17283 (10)	0.0218 (3)	
N1	0.40302 (6)	0.38813 (17)	0.34120 (11)	0.0193 (3)	
C1	0.31660 (7)	0.4152 (2)	0.45726 (14)	0.0206 (4)	
C2	0.29195 (7)	0.4945 (2)	0.53786 (14)	0.0234 (4)	
H2	0.3113	0.5258	0.5987	0.028*	
C3	0.23838 (7)	0.5274 (2)	0.52817 (15)	0.0260 (4)	
H3	0.2210	0.5839	0.5819	0.031*	
C4	0.21024 (7)	0.4782 (2)	0.44037 (15)	0.0256 (4)	
H4	0.1734	0.4989	0.4354	0.031*	
C5	0.23497 (7)	0.3991 (2)	0.35976 (15)	0.0233 (4)	
H5	0.2151	0.3656	0.3003	0.028*	
C6	0.28911 (7)	0.36857 (19)	0.36578 (14)	0.0194 (4)	
C7	0.31751 (7)	0.29795 (19)	0.27785 (14)	0.0198 (4)	
C8	0.37096 (7)	0.3116 (2)	0.26458 (14)	0.0190 (4)	
C9	0.39673 (7)	0.2439 (2)	0.17199 (14)	0.0202 (4)	
C10	0.47565 (8)	0.1946 (2)	0.08044 (15)	0.0271 (4)	
H10A	0.4664	0.0886	0.0725	0.041*	
H10B	0.5140	0.2044	0.0894	0.041*	
H10C	0.4645	0.2496	0.0169	0.041*	
C11	0.42697 (7)	0.5340 (2)	0.30952 (15)	0.0228 (4)	
H11A	0.4436	0.5221	0.2387	0.027*	
H11B	0.4554	0.5591	0.3606	0.027*	
C12	0.38804 (8)	0.6648 (2)	0.30478 (16)	0.0272 (4)	
H12A	0.3576	0.6377	0.2591	0.033*	
H12B	0.3745	0.6864	0.3770	0.033*	
C13	0.41478 (8)	0.8014 (2)	0.26092 (17)	0.0316 (5)	
H13	0.4245	0.8001	0.1880	0.038*	
C14	0.42573 (8)	0.9223 (2)	0.31568 (17)	0.0306 (5)	
H14A	0.4166	0.9276	0.3888	0.037*	
H14B	0.4428	1.0045	0.2823	0.037*	
H1O	0.3066 (11)	0.199 (3)	0.159 (2)	0.046*	

### Atomic displacement parameters (Å^2^)

**Table:**

	*U*^11^	*U*^22^	*U*^33^	*U*^12^	*U*^13^	*U*^23^
S1	0.0183 (2)	0.0232 (2)	0.0185 (2)	−0.00070 (17)	−0.00184 (16)	−0.00094 (17)
O1	0.0193 (6)	0.0285 (7)	0.0221 (7)	−0.0037 (5)	−0.0026 (5)	−0.0032 (5)
O2	0.0240 (7)	0.0246 (7)	0.0227 (7)	0.0010 (5)	−0.0039 (5)	0.0011 (5)
O3	0.0231 (7)	0.0310 (7)	0.0217 (7)	−0.0030 (5)	−0.0018 (5)	−0.0041 (6)
O4	0.0211 (6)	0.0284 (7)	0.0230 (7)	−0.0001 (5)	−0.0031 (5)	−0.0043 (6)
O5	0.0174 (6)	0.0261 (7)	0.0219 (6)	0.0018 (5)	−0.0002 (5)	−0.0021 (5)
N1	0.0171 (7)	0.0230 (8)	0.0178 (7)	−0.0020 (6)	−0.0010 (6)	−0.0018 (6)
C1	0.0194 (8)	0.0202 (9)	0.0221 (9)	−0.0026 (7)	0.0016 (7)	0.0027 (7)
C2	0.0250 (9)	0.0250 (10)	0.0201 (9)	−0.0023 (8)	0.0012 (7)	−0.0004 (7)
C3	0.0264 (9)	0.0256 (10)	0.0260 (10)	0.0012 (8)	0.0076 (7)	0.0014 (8)
C4	0.0193 (9)	0.0278 (10)	0.0297 (10)	0.0015 (7)	0.0036 (7)	0.0062 (8)
C5	0.0192 (9)	0.0267 (10)	0.0241 (9)	−0.0029 (7)	−0.0001 (7)	0.0043 (8)
C6	0.0189 (8)	0.0187 (9)	0.0205 (9)	−0.0026 (7)	0.0001 (7)	0.0028 (7)
C7	0.0200 (8)	0.0194 (9)	0.0198 (9)	−0.0010 (7)	−0.0022 (7)	0.0018 (7)
C8	0.0186 (8)	0.0194 (8)	0.0189 (9)	−0.0013 (7)	−0.0025 (7)	−0.0004 (7)
C9	0.0196 (9)	0.0196 (9)	0.0213 (9)	0.0006 (7)	−0.0008 (7)	0.0027 (7)
C10	0.0241 (9)	0.0319 (11)	0.0253 (10)	0.0034 (8)	0.0040 (8)	−0.0036 (8)
C11	0.0210 (9)	0.0243 (10)	0.0230 (9)	−0.0048 (7)	0.0010 (7)	−0.0020 (8)
C12	0.0281 (10)	0.0249 (10)	0.0286 (10)	−0.0031 (8)	−0.0032 (8)	−0.0006 (8)
C13	0.0398 (12)	0.0307 (11)	0.0242 (10)	−0.0041 (9)	−0.0020 (9)	0.0037 (9)
C14	0.0343 (11)	0.0261 (10)	0.0313 (11)	−0.0013 (8)	−0.0053 (8)	0.0042 (9)

### Geometric parameters (Å, º)

**Table:**

S1—O2	1.4346 (14)	C5—C6	1.397 (2)
S1—O3	1.4356 (13)	C5—H5	0.9500
S1—N1	1.6396 (16)	C6—C7	1.462 (2)
S1—C1	1.7561 (19)	C7—C8	1.366 (2)
O1—C7	1.343 (2)	C8—C9	1.465 (3)
O1—H1O	0.84 (3)	C10—H10A	0.9800
O4—C9	1.227 (2)	C10—H10B	0.9800
O5—C9	1.325 (2)	C10—H10C	0.9800
O5—C10	1.449 (2)	C11—C12	1.528 (3)
N1—C8	1.433 (2)	C11—H11A	0.9900
N1—C11	1.490 (2)	C11—H11B	0.9900
C1—C2	1.385 (3)	C12—C13	1.499 (3)
C1—C6	1.407 (2)	C12—H12A	0.9900
C2—C3	1.391 (3)	C12—H12B	0.9900
C2—H2	0.9500	C13—C14	1.310 (3)
C3—C4	1.386 (3)	C13—H13	0.9500
C3—H3	0.9500	C14—H14A	0.9500
C4—C5	1.385 (3)	C14—H14B	0.9500
C4—H4	0.9500		
			
O2—S1—O3	119.04 (8)	C8—C7—C6	122.63 (16)
O2—S1—N1	108.04 (8)	C7—C8—N1	121.29 (16)
O3—S1—N1	108.25 (8)	C7—C8—C9	119.96 (16)
O2—S1—C1	108.24 (8)	N1—C8—C9	118.73 (15)
O3—S1—C1	110.00 (8)	O4—C9—O5	123.51 (17)
N1—S1—C1	101.90 (8)	O4—C9—C8	122.61 (16)
C7—O1—H1O	104.9 (18)	O5—C9—C8	113.87 (15)
C9—O5—C10	115.33 (14)	O5—C10—H10A	109.5
C8—N1—C11	117.79 (14)	O5—C10—H10B	109.5
C8—N1—S1	114.29 (12)	H10A—C10—H10B	109.5
C11—N1—S1	118.72 (12)	O5—C10—H10C	109.5
C2—C1—C6	121.90 (17)	H10A—C10—H10C	109.5
C2—C1—S1	121.65 (14)	H10B—C10—H10C	109.5
C6—C1—S1	116.46 (13)	N1—C11—C12	114.66 (15)
C1—C2—C3	118.80 (17)	N1—C11—H11A	108.6
C1—C2—H2	120.6	C12—C11—H11A	108.6
C3—C2—H2	120.6	N1—C11—H11B	108.6
C4—C3—C2	120.14 (17)	C12—C11—H11B	108.6
C4—C3—H3	119.9	H11A—C11—H11B	107.6
C2—C3—H3	119.9	C13—C12—C11	110.25 (17)
C5—C4—C3	120.96 (17)	C13—C12—H12A	109.6
C5—C4—H4	119.5	C11—C12—H12A	109.6
C3—C4—H4	119.5	C13—C12—H12B	109.6
C4—C5—C6	120.12 (18)	C11—C12—H12B	109.6
C4—C5—H5	119.9	H12A—C12—H12B	108.1
C6—C5—H5	119.9	C14—C13—C12	124.9 (2)
C5—C6—C1	118.02 (17)	C14—C13—H13	117.6
C5—C6—C7	121.60 (16)	C12—C13—H13	117.6
C1—C6—C7	120.29 (16)	C13—C14—H14A	120.0
O1—C7—C8	122.81 (16)	C13—C14—H14B	120.0
O1—C7—C6	114.53 (15)	H14A—C14—H14B	120.0
			
O2—S1—N1—C8	61.69 (14)	C5—C6—C7—O1	−20.8 (2)
O3—S1—N1—C8	−168.15 (12)	C1—C6—C7—O1	162.66 (16)
C1—S1—N1—C8	−52.19 (14)	C5—C6—C7—C8	157.21 (18)
O2—S1—N1—C11	−152.15 (12)	C1—C6—C7—C8	−19.3 (3)
O3—S1—N1—C11	−21.99 (15)	O1—C7—C8—N1	−177.64 (15)
C1—S1—N1—C11	93.96 (14)	C6—C7—C8—N1	4.5 (3)
O2—S1—C1—C2	104.25 (16)	O1—C7—C8—C9	0.7 (3)
O3—S1—C1—C2	−27.34 (18)	C6—C7—C8—C9	−177.14 (16)
N1—S1—C1—C2	−142.01 (15)	C11—N1—C8—C7	−110.93 (19)
O2—S1—C1—C6	−75.07 (15)	S1—N1—C8—C7	35.6 (2)
O3—S1—C1—C6	153.34 (13)	C11—N1—C8—C9	70.7 (2)
N1—S1—C1—C6	38.67 (15)	S1—N1—C8—C9	−142.81 (14)
C6—C1—C2—C3	0.6 (3)	C10—O5—C9—O4	2.4 (3)
S1—C1—C2—C3	−178.67 (14)	C10—O5—C9—C8	−177.74 (15)
C1—C2—C3—C4	1.6 (3)	C7—C8—C9—O4	4.8 (3)
C2—C3—C4—C5	−1.7 (3)	N1—C8—C9—O4	−176.84 (16)
C3—C4—C5—C6	−0.3 (3)	C7—C8—C9—O5	−175.05 (16)
C4—C5—C6—C1	2.4 (3)	N1—C8—C9—O5	3.3 (2)
C4—C5—C6—C7	−174.17 (17)	C8—N1—C11—C12	74.1 (2)
C2—C1—C6—C5	−2.6 (3)	S1—N1—C11—C12	−70.88 (19)
S1—C1—C6—C5	176.72 (14)	N1—C11—C12—C13	−173.83 (15)
C2—C1—C6—C7	174.05 (17)	C11—C12—C13—C14	−111.0 (2)
S1—C1—C6—C7	−6.6 (2)		

### Hydrogen-bond geometry (Å, º)

**Table:**

*D*—H···*A*	*D*—H	H···*A*	*D*···*A*	*D*—H···*A*
C4—H4···O2^i^	0.95	2.52	3.269 (2)	136
O1—H1*O*···O4	0.84 (3)	1.81 (3)	2.577 (2)	151 (3)

Symmetry code: (i) −*x*+1/2, *y*+1/2, *z*.
